# Supplementary material for: Efficacy and safety of upadacitinib in the treatment of moderate-to-severe atopic dermatitis: A systematic review
Source: PLoS One. 2024 Jul 26;19(7):e0306463. doi: 10.1371/journal.pone.0306463 (PMC11280219; doi:10.1371/journal.pone.0306463)
Supplement: S1 Fig — (DOC) [file pone.0306463.s002.doc]

**Supplementary Figures:**


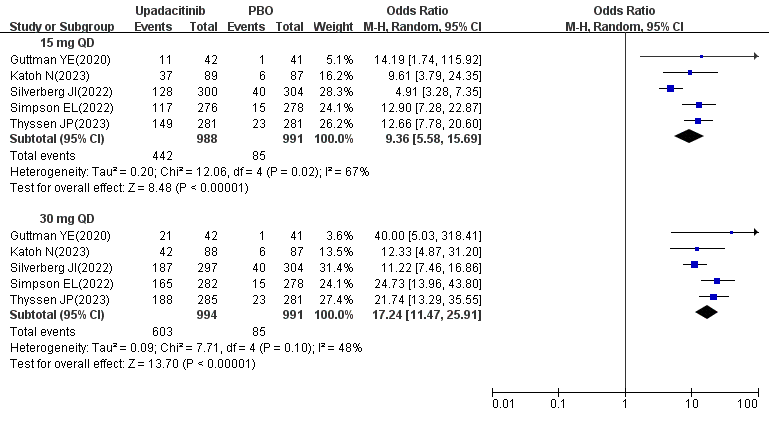

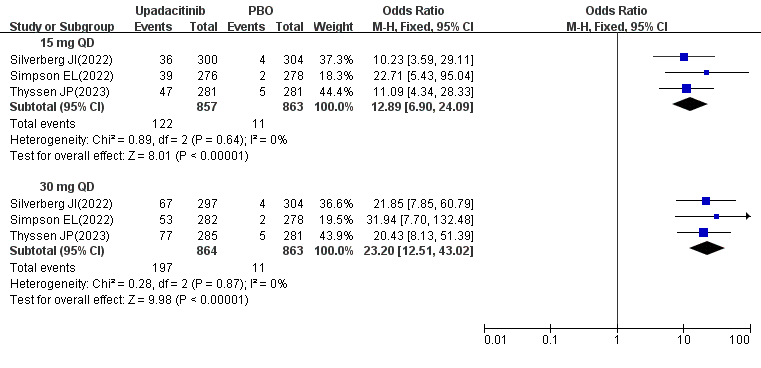


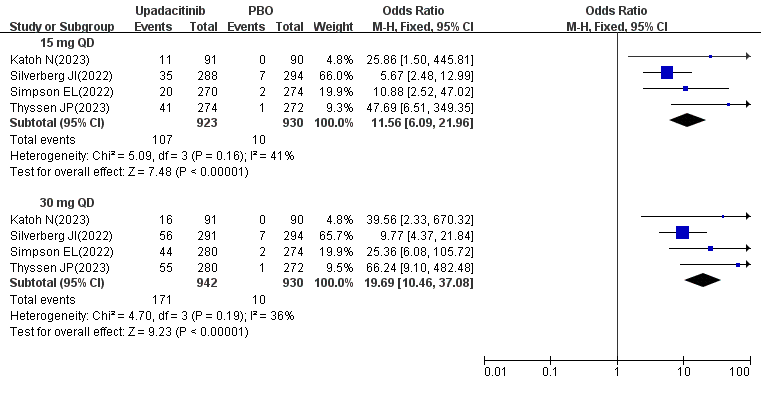

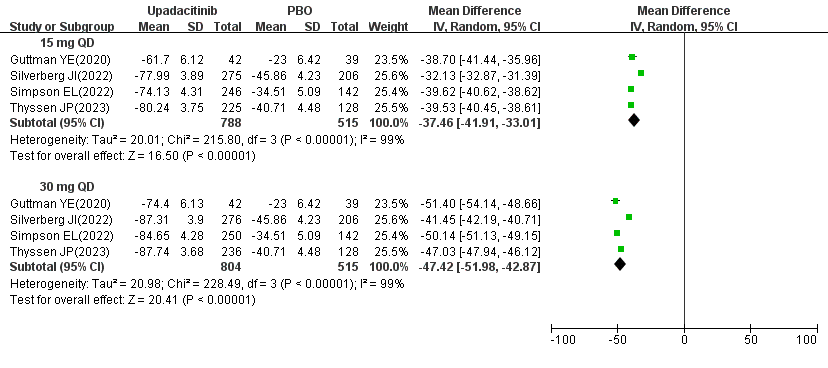


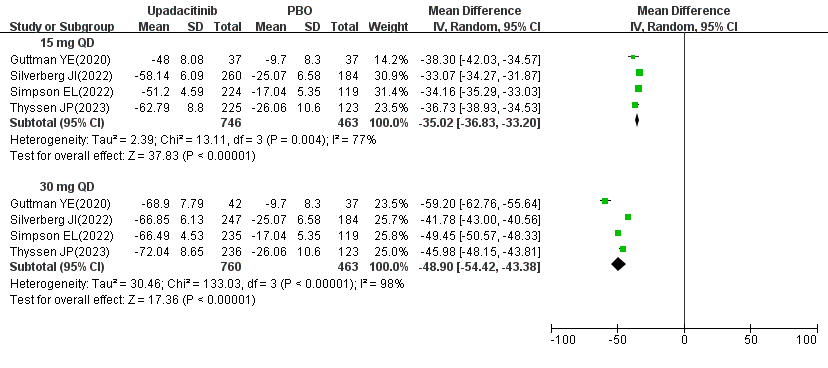

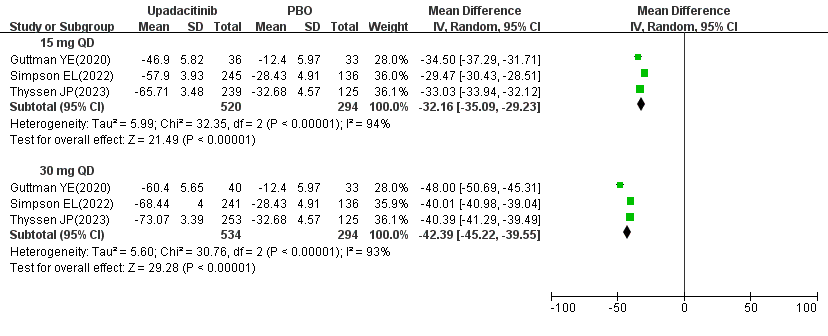


**A**

**B**

**C**

**D**

**E**

**F**

**S1 Fig. Meta-analysis forest plots of other efficacy measures.**

(A) EASI-90%; (B) EASI-100%; (C) 24h NRS ≥ 4; (D) EASI baseline score; (E) NRS baseline score; (F) SCORAD baseline score.
