# Supplementary material for: Efficacy and safety of upadacitinib in the treatment of moderate-to-severe atopic dermatitis: A systematic review
Source: PLoS One. 2024 Jul 26;19(7):e0306463. doi: 10.1371/journal.pone.0306463 (PMC11280219; doi:10.1371/journal.pone.0306463)
Supplement: S2 Fig — (DOC) [file pone.0306463.s003.doc]

**Supplementary Figures:**


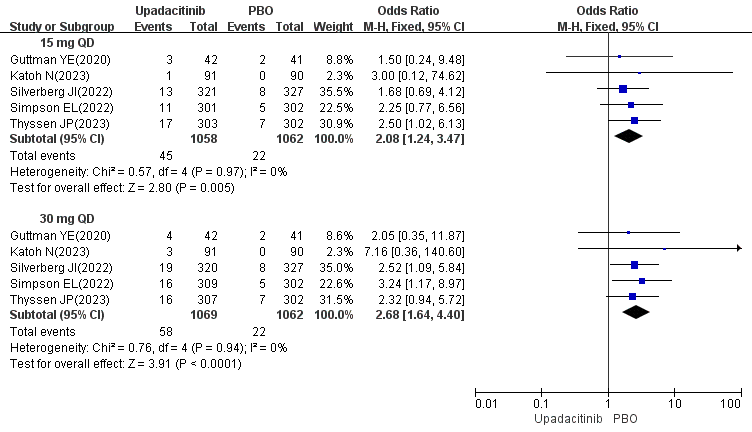

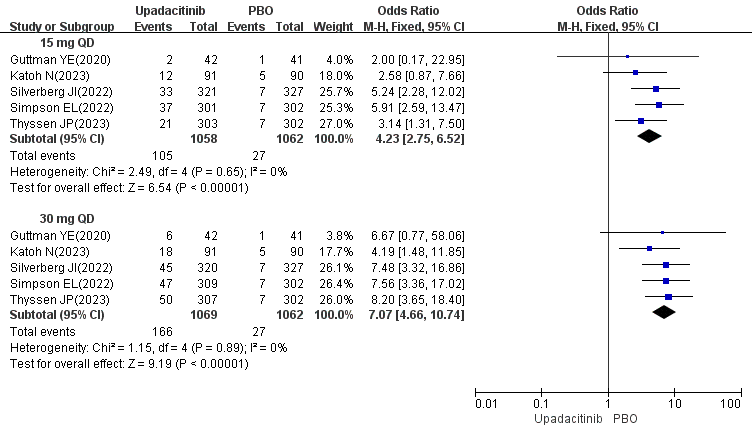


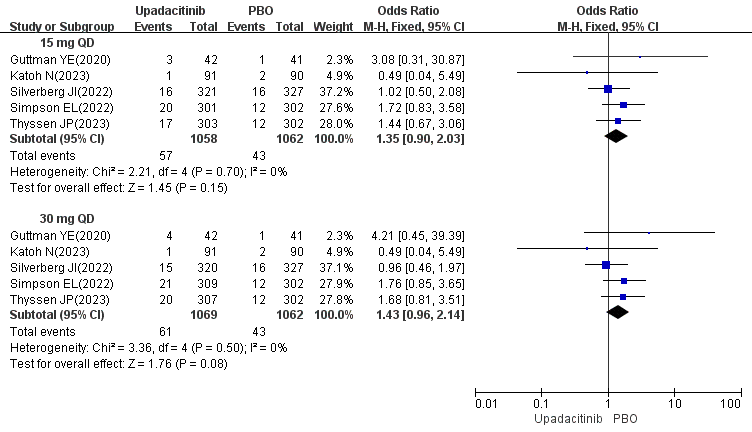

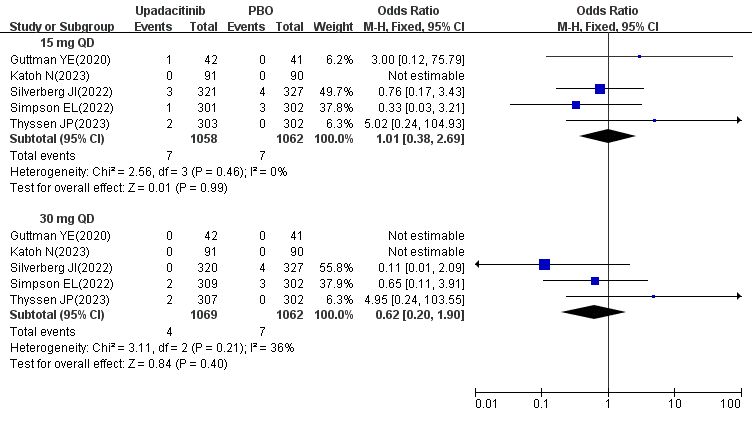


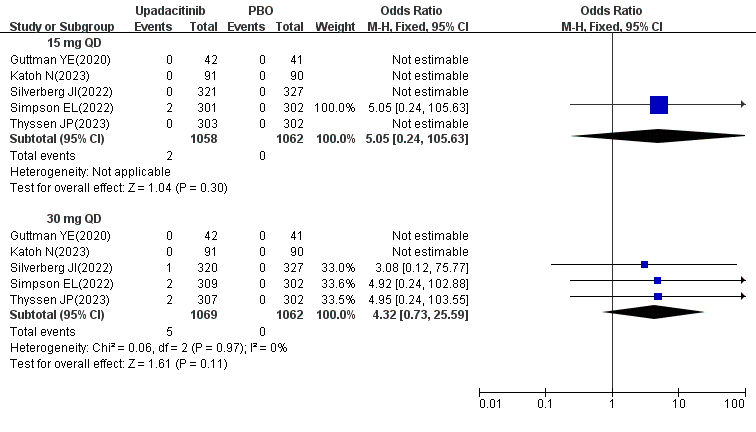

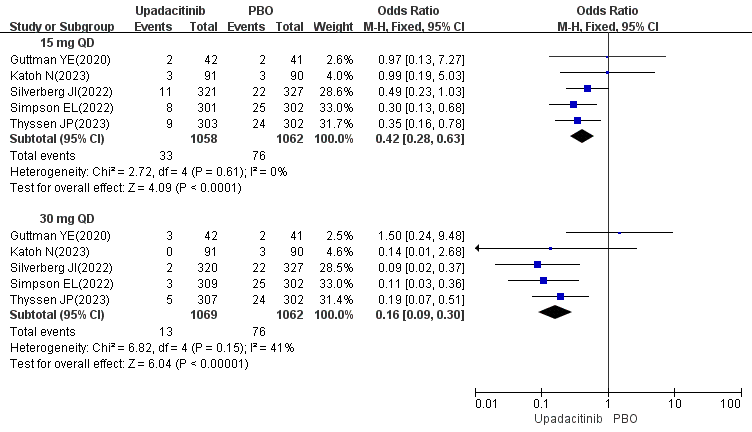


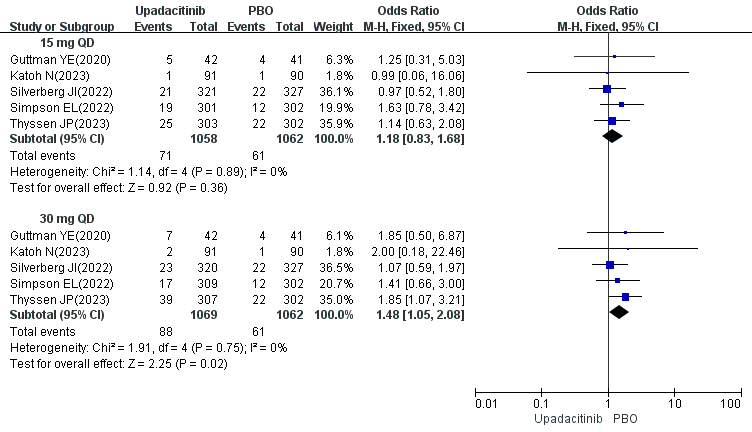

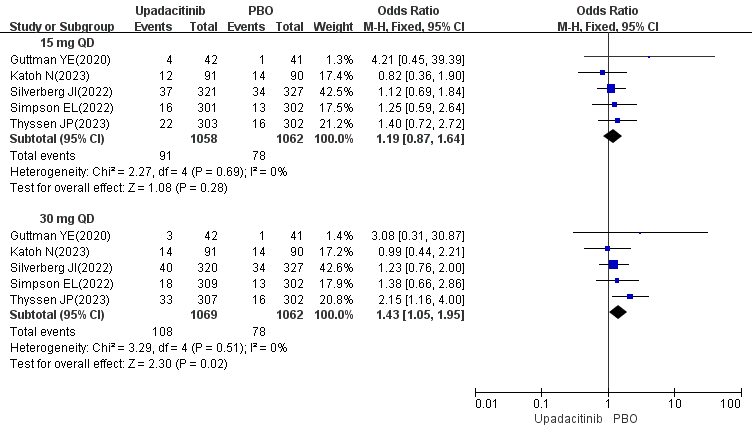


**A**

**B**

**C**

**D**

**E**

**F**

**G**

**H**

**S2 Fig. Meta-analysis forest plots of other safety measures.**

(A) Blood Creatine Phosphokinase Increased; (B) Acne; (C) Headache; (D) Serious infection; (E) Cancer; (F) Allergic dermatitis; (G) Upper respiratory tract infection; (H) Nasopharyngitis.
