# Supplementary material for: Efficacy and safety of upadacitinib in the treatment of moderate-to-severe atopic dermatitis: A systematic review
Source: PLoS One. 2024 Jul 26;19(7):e0306463. doi: 10.1371/journal.pone.0306463 (PMC11280219; doi:10.1371/journal.pone.0306463)
Supplement: Attachment — Submitted filename: review.docx [file pone.0306463.s005.docx]

PONE-D-24-11869

Good comprehensive article

My suggestion is to add frequency of adverse events .

Frequency of cancers and thrombotic events has been mentioned, please add frequency of more common side effects like headache and nasopharyngitis.
