# Supplementary material for: Efficacy and safety of upadacitinib in the treatment of moderate-to-severe atopic dermatitis: A systematic review
Source: PLoS One. 2024 Jul 26;19(7):e0306463. doi: 10.1371/journal.pone.0306463 (PMC11280219; doi:10.1371/journal.pone.0306463)
Supplement: Attachment — Submitted filename: Response to Reviewers.docx [file pone.0306463.s006.docx]

Dear Editors and Reviewers:

Thank you for your letter and for the reviewers’ comments concerning our manuscript entitled “Efficacy and safety of upadacitinib in the treatment of moderate-to-severe atopic dermatitis: A systematic review”. Those comments are all valuable and very helpful for revising and improving our paper, as well as the important guiding significance to our researches. We have studied comments carefully and have made correction which we hope meet with approval. Revised portion are marked in the paper. The main corrections in the paper and the responds to the reviewers' comments are as follows:

**Point by point reply to editors’and reviewers’ scomments:**

**Editor：**1.Please ensure that your manuscript meets PLOS ONE's style requirements, including those for file naming.

Response: Thank you for your careful suggestion. We have made revisions to the manuscript in accordance with the style requirements of PLOS ONE.

2. Please confirm at this time whether or not your submission contains all raw data required to replicate the results of your study. Authors must share the “minimal data set” for their submission.

Response: Thank you for your thoughtful suggestion. The original data mentioned in the manuscript have been compiled into a file named "Raw data" and we are willing to share the dataset.

3. Please review your reference list to ensure that it is complete and correct. If you have cited papers that have been retracted, please include the rationale for doing so in the manuscript text, or remove these references and replace them with relevant current references. Any changes to the reference list should be mentioned in the rebuttal letter that accompanies your revised manuscript. If you need to cite a retracted article, indicate the article’s retracted status in the References list and also include a citation and full reference for the retraction notice.

Response: Thank you for your careful suggestion. We have carefully checked each of these references to make sure they are complete and correct. In addition to this, we have added six references to support our argument for the efficacy and safety of upadacitinib with respect to variations in ethnicity. (references 35-40)

**Reviewer #1:** Good comprehensive article

My suggestion is to add frequency of adverse events .

Frequency of cancers and thrombotic events has been mentioned, please add frequency of more common side effects like headache and nasopharyngitis.

Response: Thank you for your affirmative and pertinent comments. According to your comments, we have added information about frequency of more common side effects in the discussion section, which has been marked in the paper. (lines 277-285)

**Reviewer #2:** To mention in detail regarding the efficacy of upadacitinib with respect to variations in ethnicity. Is it equally effective in all races? If not how does it differ? Any difference in onset of getting a therapeutic response/ duration of treatment/ side effects?

Response: Thank you for your valuable suggestion which we hadn't thought of. According to a literature review, we have launched a detailed description of the efficacy and safety of upadacitinib with respect to ethnic variations in the discussion section, which has been marked in the paper. Meanwhile, we have added six references to support our arguments. (lines 316-334; references 35-40)

**Reviewer #3:** Thank you for your work; the interest in using Upadacitinib is increasing to treat refractory and unrespondant atopic dermatitis to the (classical) therapeutic option. However, the safety profile is a crucial concern. This manuscript will provide an additional overview of using this medication.

Response: Thank you so much for your kind and encouraging comment! It would indeed be an honor for us if our research could be published in PLOS ONE.

We appreciate for Editors/Reviewers’ warm work earnestly, and hope that the correction will meet with approval.

Once again, thank you very much for your comments and suggestions.

Yours sincerely,

Peng Tang on behalf of the authors.

Corresponding author: Peng Tang at Department of TCM Pharmacy, Chengdu Integrated TCM and Western Medicine Hospital, Chengdu 610000, Sichuan, China. Email: tangpeng19940308@163.com.
